# Feedback and guiding questions as tools for reflective writing: a comparative study among undergraduate medical students in India

**DOI:** 10.1186/s12909-025-07767-7

**Published:** 2025-08-18

**Authors:** Shruti Prabhat Hedge, Vijay Kautilya Dayanidhi

**Affiliations:** 1 Department of Ophthalmology, Manipal TATA Medical College-Jamshedpur, Manipal Academy of Higher Education (MAHE), Manipal, India; 2 Department of Forensic medicine & Toxicology, Manipal TATA Medical College-Jamshedpur, Manipal Academy of Higher Education (MAHE), Manipal, India

**Keywords:** Reflective writing, Guiding questions, Feedback, Competency based medical education

## Abstract

**Introduction:**

Reflective writing helps foster clinical reasoning, and professional development in medical students. This study attempts to compare the effectiveness of two interventions—Structured guiding questions and individualized feedback for enhancing reflective writing skills among undergraduate medical students in India.

**Methodology:**

A quasi-experimental, semi-qualitative study was conducted among 30 third-year MBBS students of Shri Sathya Sai Medical College and Research Institute, Chennai. Participants were randomly allocated into two groups of 15 each. Group A used a structured guiding question, while Group B received individual feedback on the reflections from trained faculty. All students wrote five reflections pre- and post-intervention on their clinical experiences in Ophthalmology. The fifth reflections were assessed by three experts using a validated rubric. Pre- and post-intervention scores were compared using the Wilcoxon Signed Rank Test, and Mann-Whitney U Test was used for intergroup comparison. Semi-quantitative feedback and open-ended responses were used for student perception.

**Results:**

Both interventions led to statistically significant improvements in reflective writing (*p* < 0.01). Guiding question Group’s average score improved from 1.33 to 2.27, and Feedback Groups from 1.13 to 2.00. No significant difference was observed between the post-intervention scores of both groups (*p* > 0.05). Feedback and guiding questions, both were perceived to be useful, as guiding questions helped structure the reflection, and feedback supported emotional connection.

**Conclusion:**

Both tools were found to be effective to improve the quality of reflections. Integrating guiding questions and feedback can prove to be a better strategy to support Competency Based Medical Education.

**Trial registration:**

‘Clinical trial number not applicable.’

**Supplementary Information:**

The online version contains supplementary material available at 10.1186/s12909-025-07767-7.

## Introduction

Medical education in India is undergoing a radical change with the introduction of competency-based medical education. There is a shift towards a competency-based model for curriculum design rather than a subject-based model in the traditional curriculum. With the adoption of this student-centered curriculum, the emphasis on achievement of the competencies is the core principle driving the process of curriculum design [18, 22]. This transformative approach aims to equip medical graduates with the essential skills and knowledge to address the evolving healthcare needs of society [12]. - The medical education curriculum in India, as set by the National Medical Council, emphasizes self-directed learning and continuous professional development [8, 16]. There has been a persistent call for reforms in the curriculum that will cater to the community’s needs [23]. - With the emphasis on competency acquisition, it becomes necessary that self-reflection become an integral part of medical education [15]. - Reflection and feedback are metacognitive strategies that enhance the learning process, playing a vital role in promoting deep and meaningful learning. Reflection has been consciously introduced into the curriculum so that learning is enhanced and internalized by the students. Reflection encourages students to analyse their experiences, identify areas for improvement, and develop strategies to enhance their future performance.

Reflective writing is a relatively new concept in undergraduate medical education in India. Culturally, we are not accustomed to expressing ourselves, and a reflective writing assignment may seem alien to many students. Students find it difficult to express themselves freely and openly in the written format. Although reflections have been introduced as a routine component in student logbooks, many educators in the Indian medical education context observe that the depth and quality of these reflections often vary and may not meet the intended educational goals. In light of this background, it becomes critical to investigate how reflection can be inculcated into the learning process. Medical educators across the globe are increasingly recognizing the importance of reflection as a critical skill for training physicians who can creatively respond to complex health systems, clinical cases, and social situations [2]. Reflective learning is considered an important component of self-regulation and continuous professional development. While considerable literature exists on the theoretical underpinnings of reflective practice, there is a relative lack of empirical studies examining effective strategies for teaching and facilitating reflection in medical education [2, 24]. (.

Reflective writing is a purposeful activity requiring the student to consciously analyze his experience to draw conclusions about what happened, what the outcome of the actions was, and what needs to be done to better the same [20, 24]. - Effective reflections require the students to sequence their thoughts purposefully to answer the above questions. Providing them with a format or a guide for reflective writing can be effective to facilitate this process. Guiding question formats can serve as prompts to help students to delve deeper into their experiences and articulate their thoughts more effectively. They are meant to provide the student a framework or a structure for effective reflection by directing their attention to specific aspects of their experience. They may in principle serve as an excellent starter tool to help students learn the art of purposeful and directed reflective writing [1, 2, 9, 14].

Guiding questions can also prompt the students to delve deeper into their experiences and articulate their thoughts effectively. They can encourage analysis of the experiences, identify core areas of improvement, and promote critical thinking. They can help to explore the emotional aspects of the experience while encouraging critical thinking to form individualized learning strategies. By prompting students to consider different perspectives, challenge assumptions, and explore alternative solutions, guiding questions can foster a more comprehensive understanding of their experiences. In all, guiding questions can prove to be effective to structure and improve the quality of reflective writing among beginners [6, 14, 25].

Feedback is an equally important component of reflective writing. Feedback in reflective writing provides learners with valuable insights into their strengths and weaknesses, helping them to refine their reflective skills and deepen their understanding of the material. Constructive feedback can help learners identify areas where they may be struggling or overlooking important details while also reinforcing positive aspects of their reflection. Feedback can be personalized and tailored to each individual student, and every time feedback is given by a faculty member or mentor, it tends to improve specific areas of student reflection, building on the previous suggestions. It is more realistic and a continuous process and helps to monitor students’ improvements. It can serve as an assessment of the students’ abilities to reflect and make calculated suggestions to target weak areas of a student’s reflection [1, 9, 14, 20].

Feedback can prompt students to challenge their views and adopt alternative views. It can help learners focus on validated indicators of competency development and remain motivated towards purposeful reflection. Feedback is an effective tool for mentoring and guiding the student in the whole process of reflective learning. Feedback provided on the reflections focusing on the structure of the writing rather than the content has been shown to improve the quality of reflections [14]. The combined effect of structured reflective writing with directed feedback from a mentor or a group has been shown to be the most effective teaching method for enhancing a student’s reflective capacity [26]. Feedback, when integrated with reflective writing, can address gaps in the performances that reflection alone may not identify. It can help the learner to set goals, monitor his progress, and adjust his thoughts to achieve the desired goal. Constructive feedback helps students identify areas they are struggling and overlooking [3]. Feedback tends to be personalized and addresses the needs of the student [13].

The significance of feedback and reflection in medical education aligns with self-regulated learning theory, highlighting their role in enhancing self-regulation, deepening understanding, and improving clinical competency [11].

Significant data is available on the role that guiding questions and feedback individually play in promoting reflective writing, but a comparative analysis of the two is lacking [11]. Cultural and regional factors have a significant influence on the learning styles and preferences of students, and the effectiveness of educational interventions can vary across different cultural contexts. Therefore, there is a need for context-specific research to inform the design and implementation of effective strategies for promoting reflective writing among undergraduate medical students in India.

This study aims to compare the effectiveness of guiding questions and feedback as tools for promoting reflective writing among undergraduate medical students in India. This study will provide valuable insights into the relative effectiveness of these two approaches in the Indian context and inform the development of evidence-based strategies for promoting reflective writing in medical education. The research will add to the existing literature by providing empirical evidence on the comparative effectiveness of guiding questions and feedback in promoting reflective writing, specifically within the context of undergraduate medical education in India.

## Methodology

### Study Design and Setting:

A quasi-experimental, semi-qualitative interventional study was conducted at Shri Sathya Sai Medical College and Research Institute, Chennai, among third-year MBBS students. The study was carried out among third-year MBBS students between September 2020 and March 2021 as part of a curriculum-based educational intervention.

### Participants

The study was conducted among 30 third-year MBBS students during their clinical postings in the Department of Ophthalmology. Convenience sampling was adopted. All 30 students from the selected batch were approached, and all of them voluntarily agreed to participate and provided written informed consent.

### Developing the guiding question format and reflective assessment rubric:

A panel of 10 faculty, comprising members of the Medical Education Unit (MEU) and clinical faculty, participated in designing the components/tools of the study. The following tools were developed for the study.

- A structured format with guiding questions was developed to aid students in reflective writing after literature review and deliberation. The format consisted of seven questions to be answered sequentially to complete the reflections. The same was face validated by the panel of experts for relevance, comprehensiveness, and clarity. Table No 1 provides the details of the guiding questions.

**Table 1:** Guiding Questions for Reflective Writing

WHAT (What happened?)	1. What was the most significant thing you learned today, and how has it influenced your perspective or approach? 2. What experience or observation this week surprised you the most, and why? 3. What aspect of your clinical experience this week was most disappointing or challenging, and what did you learn from it?
SO, WHAT (Why does it matter?)	4. How do you plan to apply this new knowledge or skill in your future clinical practice? 5. If you could change one aspect of your clinical work or learning experience, what would it be and how would you improve it?
NOW WHAT (What will you do next?)	6. What potential obstacles might you face in implementing this change, and how could you overcome them? 7. What specific area or skill do you plan to explore or develop further based on your experiences this week?

- Reflection Assessment Rubric: A novel assessment rubric was designed to assess the quality of reflective writing. The rubric consisted of a 4-point Likert scale to grade the components of the reflective writing like reflective ability, critical analysis and making connections. The same was validated for its clarity, relevance and comprehensiveness.
- Feedback forms for student perception on the usefulness of the feedback and guiding question were developed and validated for by the panel of experts.

### Intervention

The intervention was carried out in a phased manner over six months in the department of Ophthalmology during the clinical postings as described in Figure no 1. The 30 participants were initially briefed about reflective writing and then asked to write reflections on their clinical experience/encounters during the clinical posting. Then they were asked to write 5 such reflections before the actual intervention. Participants were randomly divided into two groups of 15 each by lottery method. Group A was given a structured format with guiding questions, and Group B was given detailed feedback on their reflections from faculty trained in reflective practice. Each reflection was written over 20–30 min at the end of the clinical day. The students in group A wrote 5 reflections on their clinical encounters using only the guiding questions and Students in Group B wrote five reflections based on their clinical experience and then received feedback on their reflections from faculty trained in reflective practice and medical education. Faculty provided constructive criticism, identified strengths and weaknesses, and suggested areas for improvement. The feedback included comments on clarity, emotional depth, critical thinking, and suggestions for improvement. The fifth reflection before the intervention and 5th reflection after the intervention in both the groups were assessed using the reflective assessment rubric by three experts independently. The 5th reflection was used to allow students time to become familiar with the intervention method, thereby improving the quality and depth of the final reflection. It also ensured a standardized point of comparison and reduced rater fatigue. The average score was taken for the final analysis. Student feedback was taken on the usefulness of the guiding questions and feedback for improving reflective writing.

**Fig. 1:**
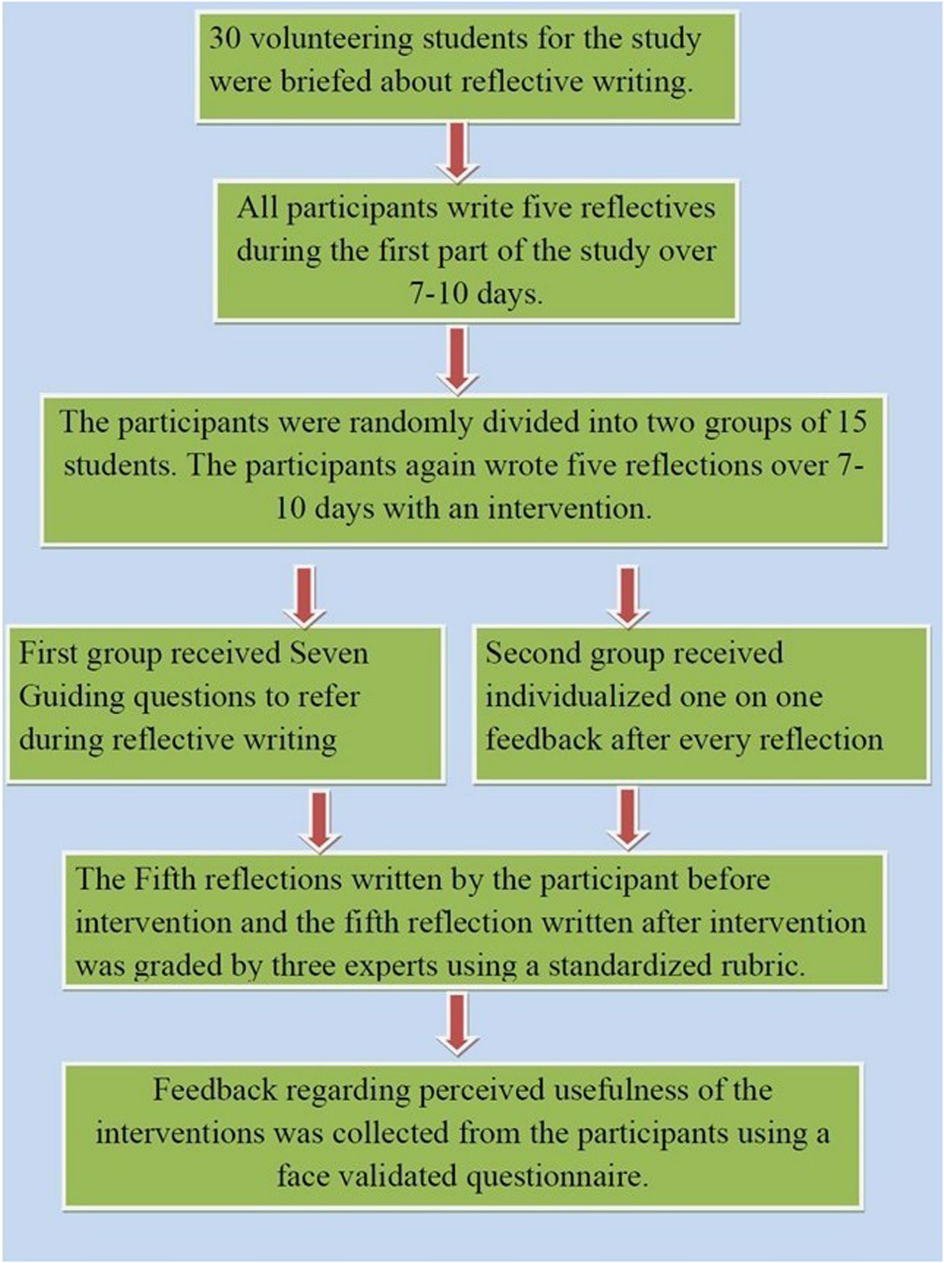
Study design depicting the study phases

## Data collection and analysis:

### Quantitative data:

#### Pre and post-intervention reflection analysis

The scores obtained by the students before the intervention were compared with the scores obtained after intervention using the non-parametric Wilcoxon test. The scores obtained by the Group A (Guiding Questions) was compared with Group B (Feedback) using the Mann Whitney U test.

The perceived usefulness and efficacy of the intervention tools were assessed using a semi-quantitative feedback questionnaire administered to the students in both groups. The responses were compiled and analysed using descriptive statistics, and the results were visually represented.

#### Qualitative analysis

Thematic analysis of students’ responses to open-ended questions was conducted to identify recurring themes and patterns related to their experiences with reflective writing, the usefulness of feedback, and the impact of guiding questions on their learning.

All statistical analyses were conducted using *Jamovi open-source statistical software* (version 2.6.26). A p-value of less than 0.05 was considered indicative of statistical significance.

## Results

The study participants comprised 27%(*n* = 8) male and 73%(*n* = 22) female students, with a mean age of 23.4 years.

### Pre- and post-intervention reflection analysis

#### Guiding question group

From the comparison of the pre-intervention and post-intervention scores of the reflections in the Group A with Guiding questions it can be observed that the average post-intervention score was 2.27 compared to the pre-intervention average of 1.33. Individual component mean scores in the rubrics post-intervention for reflective thinking, quality of analysis and making connections was 2.47, 2.27 and 2.07 respectively compared to pre-intervention scores of 1.33, 1.40 and 1.27 showing improvement in the post-scores. Overall, 66.7% of the pre-intervention reflections were marked below average compared to 13.3% in the post-intervention. 40% of the post-intervention scores were rated to meets expectations.

#### Feedback group

From the comparison of the pre-intervention and post-intervention scores of the reflections in the Group A with Guiding questions it can be observed that the average score after intervention was 2.00 compared to the pre-intervention average of 1.13. Individual component mean scores in the rubrics post-intervention for reflective thinking, quality of analysis and making connections was 2.13, 2.00 and 2.00 respectively compared to pre-intervention scores of 1.33, 1.00 and 1.13 showing improvement in the post-scores. Overall, 86.7% of the pre-intervention reflections were marked below average compared to 20% in the post-intervention. 20% of the post-intervention scores that were rated to meets expectations.

Table No 2 depicts the results of comparison of the pre- and post-intervention reflection analysis for both the groups using Wilcoxon Signed Rank test. The results indicate a statistically significant improvement in the quality of reflections in both groups.

**Table 2:** Comparison of the Pre-interventions and Post-interventions scores:

Comparison of pre-Feedback and post-Feedback scores				
Criteria	Mean score Pre-feedback	Mean score Post- feedback.	Z score	Significance (P Value)
Reflective Thinking	1.33	2.13	-2.585	P<0.01
Analysis	1.00	2.00	-3.217	P<0.001
Making Connections	1.13	2.00	-2.919	P<0.004
Average Score	1.13	2.00	-3.357	P<0.001
Comparison of pre-and post-Guiding question (GQ) scores.				
Criteria	Mean score Pre-GQ	Mean score Post-GQ	Z score	Significance (P Value)
Reflective Thinking	1.33	2.47	-3.314	P<0.001
Analysis	1.40	2.27	-2.919	P<0.004
Making Connections	1.27	2.07	-2.640	P<0.008
Average Score	1.33	2.27	-2.889	P<0.004

#### Comparison of post-intervention scores of both the intervention groups

The scores obtained by the students after the intervention was compared using the non-parametric Mann- Whitney U test. From this data (Table no 3) it can be concluded that there was no significant difference in the scores obtained in various parameters among both the feedback and guiding questions group.

**Table 3:** Comparison of the post-intervention scores of both the intervention groups

Comparison of post-guiding question (GQ) and post-feedback scores.					
Criteria	Mean rank Post-Feedback	Mean rank Post- GQ	Z score	Significance (P Value)	Mann- Whitney U
Reflective Thinking	14.7	16.93	−1.041	*P* < 0.389	91
Analysis	13.9	17.1	−1.101	*P* < 0.325	88.5
Making Connections	15.23	15.77	−0.177	*P* < 0.870	108.5
Average Score	13.9	17.1	−1.101	*P* < 0.325	88.5

#### Analysis of feedback obtained on usefulness of the one-on-one feedback and guiding questions:

Feedback on usefulness of the intervention was obtained using a face validated questionnaire. The results of the same are tabulated in Table no 4.

**Table 4:** Feedback on usefulness of the intervention

Perception of feedback given during the reflective writing					
Feedback Question	Strongly Agree	Agree	Neither Agree nor Disagree	Disagree	Strongly Disagree
The feedback given was clear and helpful in improving the reflection.	13.3%	66.7%	20%	0%	0%
Learners understood what they will be assessed for or what ideal reflective writing is.	26.7%	46.7%	26.6%	0%	0%
Feedback focused on the task rather than the individual	26.7%	60%	13.3%	0%	0%
Feedback presented future targets which were clear, specific and achievable	60%	40%	0%	0%	0%
Feedback was aimed at motivating the learner intrinsically	26.7%	73.3%	0%	0%	0%
Perception of Guiding questions given during the reflective writing					
Feedback Question	Strongly Agree	Agree	Neither Agree nor Disagree	Disagree	Strongly Disagree
Guiding Questions given were clear and helpful to improve the reflection.	26.7%	46.7%	26.6%	0%	0%
Learners understood what they will be assessed for or what ideal Reflective writing is.	53.3%	40%	6.7%	0%	0%
Guiding questions helped to sequence the thought process during reflective writing.	20%	66.7%	13.3%	0%	0%
Guiding questions presented future targets which were clear, specific and achievable	6.7%	60%	33.3%	0%	0%
Guiding questions were aimed at motivating the learner intrinsically	20%	53.3%	26.7%	0%	0%

Qualitative feedback collected with the open-ended questions were analysed and the main themes emerging. The students felt that the one-on-one feedback was very helpful in improving their reflective writing skills. All participants felt that feedback on the reflections helped the student to evaluate and relate to their feelings. 40% of the students in the guiding question group felt that the guiding questions provided a sequence for the thoughts and helped to critically think about the experience. However, 60% of the students felt that the guiding questions were not specific to the situation and hence were difficult to answer at times.

## Discussion

Competency Based Medical Education in India aims to harbour certain set of abilities in the medical graduates that allow them to contribute to the national health goals by fulfilling certain roles in the community. Competency acquisition is a continuous process and not an event. It requires a continuous cycle of activities involving planning, gaining experience and refection on the experience in order to maximise learning [11]. We begin as a novice and evolve through different stages to become an expert through constant reflection and modification of learning strategies. Being a lifelong learner not only means to acquire new knowledge and skills it also means to keep refining skills one has already acquired. Reflective practice is now considered a core competency for healthcare professionals, fostering continuous learning and improvement [19]. Though reflection is a natural process triggered by experience in any individual, one has to take efforts to consciously make it a practice to become a reflective practitioner [20]. The reflections for learning can’t be random and hence have to be purposive and directed towards learning [7]. Medical education should hence incorporate various tools to make reflection more effective and useful for the students [10].

The study aimed to compare the effectiveness of feedback and guiding questions as tools to enhance reflective writing among undergraduate medical students. The reflections written by the students before and after the intervention were grade and compared using a pre-validated reflection rubric.

Both interventions—guiding questions and individual feedback—led to statistically significant improvements in the quality of reflective writing, as demonstrated by higher average scores in post-intervention reflections and Wilcoxon Signed Rank test results. This supports the hypothesis that structured facilitation enhances students’ reflective capacity. In the guiding question group, it can be noted that Guiding questions significantly improved the student’s reflective ability as only 13% of the post-intervention reflection scores were below average compared to 66.7% of the pre-intervention scores. Students in the Feedback group also showed similar improvement with only 20% being rated below average post-intervention compared to 86.7% pre- intervention. 40% of the post-intervention scores in guiding question group were marked to meet expectations compared to 20% in the feedback group.

The group provided with guiding questions showed a greater mean improvement in overall reflection scores (from 1.33 to 2.27) compared to the feedback group (from 1.13 to 2.00). It can be noted that “Reflective thinking” component has the most significant gain suggesting that the guiding questions might have peculiarly improved critical self-awareness. This is in alignment with other studies affirming that structured reflective exercises are better at helping students analyse their experiences and draw conclusions [11]. This also proves that guiding questions can facilitate deeper engagement with personal experiences, facilitating improved critical thinking and self-awareness. The framework for thought provided by the guiding questions is very useful for beginners for reflective writing. The questions help guide the students to probe further into their feelings, assumptions and biases [14]. Students can be motivated to move away from superficial descriptions to more meaningful engagement and analysis [14].

Contrary to this, the feedback group has exhibited a more balanced improvement across all components of reflective writing. This included description, reflective thinking, making connections and deeper analysis of their feelings. This emphasises that feedback supports holistic development of reflective skills. It can be inferred that students in the feedback group are able to understand specific areas of improvement in their writing. It encourages them to work on specific areas which may result in balanced improvement across all components of reflective writing. This finding highlights the value of personalized feedback as it can refining various facets of students’ reflective [17]. Feedback tailored to suit individual needs specifically targets to addresses weaknesses and reinforces strengths of the student, leading to a more comprehensive enhancement of reflective capabilities. Feedback every time is unique and can target a different component of reflection thus supporting uniform development.

With feedback students can better relate to the different levels of reflection thus influencing their quality of writing. Faculty need to be conscious that the feedback should be limited to the process of reflection and not the content in order to support higher order thinking [14]. The language and the sequence of thought is of limited value when considering feedback unlike the guided question format. Feedback every time provides the students with actionable elements leading to rapid improvement [27]. The findings of this study strongly support that both guiding questions and feedback can support improve student reflections.

Comparing the pos-test scores of the reflections in the guiding question and feedback group it was revealed that there was no significant difference between the groups. Despite the differences in mean improvements, Mann-Whitney U test showed no statistically significant difference between the post-intervention scores of the two groups. This suggests that both methods are comparably effective in enhancing reflection when used systematically.

From these findings it can be considered that an integrating feedback and reflections together into educational practice can improve the cognition and learning outcomes among students as noted in other studies [11].

The qualitative data collected to evaluate the perceived usefulness of the interventions reiterate that both the methods were useful. In both groups a large majority agreed or strongly agreed that the interventions were helpful and motivational. All students in the feedback group felt the feedback motivated them intrinsically, compared to 73.3% in the guiding questions group. These findings are consistent with other studies that have reported that personalized feedback improves self-regulated learning and critical assessment skills [11]. 73.4% of the students in the feedback group felt that it helped them to understand what an ideal reflection was compared to 93.3% in the guiding questions group.

Thematic analysis of the qualitative data suggests that while guiding questions helped structure thought process, some students found them too generic and not applicable in all situations. Students in guiding questions group felt that the questions pushed them to think in all possible aspect of the topic. 60% of the students felt that the guiding questions were not specific to the situation and hence were difficult to answer at times. In contrast, personalised feedback helped to bring out students’ emotions and critically evaluate their reflections.

Though both guiding questions and feedback helped to enhance reflective writing, their functional mechanism is different. Guiding questions provide a structured framework and feedback provides personalized insights. Guiding questions were helpful for students, especially those new to reflective writing, as they prompted them to explore feelings, assumptions, and bias. They enhance critical self-awareness as they guided students towards deeper engagement with personal experiences, fostering critical thinking. However, some students found them to be too generic as also reported in other studies [25].

Feedback offers a personalized insight, leading to comprehensive enhancement of reflective capabilities. A tailored approach ensures that students receive guidance that is relevant to their individual needs and learning styles [11]. It encourages students to consider different viewpoints and positively influences the quality of the written reflections [14]. The effectiveness of feedback depends on its quality, specificity, and timely delivery. Feedback when given immediately after the reflection works better.

This comparative analysis highlights that both guiding questions and feedback have unique strengths to support reflective writing among undergraduate medical students. While guiding questions offer structure and promote comprehensive exploration, feedback provides personalized insights and targeted guidance. This consistent with what is reported in similar other studies [5, 21].

The findings in our study suggest that both interventions are valuable tools, but their effectiveness may depend on individual learning preferences and each specific educational context. Both methods are not universally applicable. Integrating both approaches may potentiate a holistic strategy for promoting reflective practice among medical students by striking a balance. Faculty may consider using a blended approach, combining structured guiding questions with individualised feedback to cater to diverse learner needs. Reflective journals can be used as an effective way to develop reflective practice in higher education [4]. The study is limited by its small sample size, short duration of exposure to intervention and single-centre design. Future research could explore long-term retention of reflective skills, effectiveness across specialties, and integration of both interventions for synergistic impact.

## Conclusion

This study demonstrates that the individualised feedback and guiding questions both significantly improved the quality of the reflective writing. While guiding questions helped improve the structure and critical thinking, feedback promoted emotional engagement and self-awareness. No statistical difference was noted among the mean post-scores in both the groups. A blended approach integrating both feedback and guiding questions can offer a more comprehensive strategy to facilitate reflective writing skills. Further studies are needed to study the long-term impact and scalability.

## Supplementary Information

Supplementary Material 1.

## Data Availability

Datasets generated and/or analyzed during the current study are available from the following DOI: http://doi.org/10.6084/m9.figshare.28916174.
