# Supplementary material for: Feedback and guiding questions as tools for reflective writing: a comparative study among undergraduate medical students in India
Source: BMC Med Educ. 2025 Aug 18;25:1169. doi: 10.1186/s12909-025-07767-7 (PMC12359732; doi:10.1186/s12909-025-07767-7)
Supplement: Supplementary file 1 — Supplementary Material 1. [file 12909_2025_7767_MOESM1_ESM.docx]

Rubric for grading the Student reflections.

|  | \| Above Expectations \|  \|  \|  \| \| --- \| --- \| --- \| --- \| \|  \|  \|  \|  \| | Meets  Expectations | Approaching Expectations | Below Expectations |
| --- | --- | --- | --- | --- | --- | --- | --- | --- | --- | --- | --- | --- |
|  | 4 | 3 | 2 | 1 |
| \| **Reflective Thinking** \| \| --- \| | \| The reflection explains the student’s own thinking and learning processes, as well as implications for future learning. \| \| --- \| | \| The reflection explains the student’s thinking about his/her own learning processes. \| \| --- \| | \| The reflection attempts to demonstrate thinking about learning but is vague and/or unclear about the personal learning process. \| \| --- \| | \| The reflection does not address the student’s thinking and/or learning. \| \| --- \| |
| \| A**nalysis** \| \| --- \| | \| The reflection is an in-depth analysis of the learning experience, the value of the derived learning to self or others, and the enhancement of the student’s appreciation for the discipline. \| \| --- \| | \| The reflection is an analysis of the learning experience and the value of the derived learning to self or others. \| \| --- \| | \| The reflection attempts to analyze the learning experience but the value of the learning to the student or others is vague and/or unclear. \| \| --- \| | \| The reflection does not move beyond a description of the learning experience. \| \| --- \| |
| \| **Making Connections** \| \| --- \| | \| The reflection articulates multiple connections between this learning experience and content from other courses, past learning, life experiences and/or future goals. \| \| --- \| | \| The reflection articulates connections between this learning experience and content from other courses, past learning experiences, and/or future goals. \| \| --- \| | \| The reflection attempts to articulate connections between this learning experience and content from other courses, past learning experiences, or personal goals, but the connection is vague and/or unclear. \| \| --- \| | \| The reflection does not articulate any connection to other learning or experiences. \| \| --- \| |
